# Professor Niels Henry Secher: Celebrating success from boat to bench to bedside

**DOI:** 10.1113/EP091460

**Published:** 2023-08-30

**Authors:** Christian Aalkjær, Mads Fischer, Damian M. Bailey

**Affiliations:** ^1^ Department of Biomedicine Aarhus University Aarhus C Denmark; ^2^ Danish Cardiovascular Academy Aarhus C Denmark; ^3^ Department of Nutrition, Exercise and Sports University of Copenhagen Copenhagen Denmark; ^4^ Neurovascular Research Laboratory, Faculty of Life Sciences and Education University of South Wales Glamorgan UK

Professor Niels H. Secher, one of our outstanding modern‐day physiologists who has contributed significantly to the Copenhagen School of Exercise Physiology and control of cerebral blood flow, was the proud recipient of the Lifetime Achievement Award from the Danish Cardiovascular Academy. The award is given each year at the Danish Cardiovascular Academy summer meeting to a scientist who over a lifetime has inspired researchers in the cardiovascular field, extending the current body of knowledge.

The tradition of exercise physiology in Copenhagen was initiated by Professor August Krogh over a century ago. Krogh received the Nobel Prize in 1920 for his seminal work on capillary physiology (Krogh, 1919a, 1919b), more specifically, capillary recruitment during exercise that continues to stimulate debate in *Experimental Physiology* (Poole et al., 2013). Krogh collaborated with Professor Johannes Lindhardt, also from Copenhagen, and three of their students, who later became professors of exercise physiology in Scandinavia, quickly establishing a tradition and academic foothold. Later, Professor Bengt Saltin and colleagues continued the tradition both in Stockholm and Copenhagen, and it was this setting that attracted Niels H. Secher to start a scientific career focused on exercise physiology to compliment his clinical endeavours as a consultant anaesthesiologist.

The sheer volume and quality of exercise physiology research emanating from Copenhagen over the last century testifies to the importance of fostering supportive, collaborative environments where researchers can be creative and flourish. This is sometimes forgotten, the oil for the fire! There is another reason why Niels H. Secher became interested in exercise physiology – in his younger years, he competed in the 1968 and 1972 Olympic Games for Denmark and won the double sculls in the 1970 World Rowing Championships with Jørgen Engelbrecht (Bailey et al., 2023). Indeed, his first papers focused on the cardiopulmonary effects of rowing, with his very first paper entitled ‘Maximal oxygen uptake during arm cranking and combined arm plus leg exercise’ (Secher et al., 1974) and 2 years later, he, along with his colleague Roger Jackson, an Olympic gold medallist from Canada in 1964, published the pioneering paper on aerobic demands during rowing with data from pulmonary gases collected 'real‐time' during on water rowing (Jackson & Secher, 1976). He continues to publish on the physiology of rowing and one of his latest papers from last year was an editorial entitled ‘Advances in rowing physiology’ (Volianitis et al., 2022). His background as an elite sportsman was no doubt one of the catalysts that inspired him to study exercise physiology.

Niels H. Secher has published on many aspects of cardiovascular physiology spanning from questions that are directly relevant for the management of patients in the emergency ward including how central blood volume is best monitored and maintained, to how different anaesthetics impact haemodynamic function, even extending to how giraffes handle the orthostatic challenges imposed by gravity. Since his first publication in the early 1970s and with up to 20 papers published last year alone, his total output currently stands in excess of 700 papers. To say he has been productive is an understatement, averaging 12 papers/year over the past 50+ years! His most highly cited paper is entitled ‘Evidence for a release of brain‐derived neurotrophic factor from the brain during exercise’ published in 2009 in *Experimental Physiology* (Rasmussen et al., 2009) – a paper that received no less than 54 citations last year alone. This paper also points to the main interests in his career, namely the understanding of how cerebral perfusion is controlled during anaesthesia and in response to the haemodynamic challenges posed by postural shifts and exercise.

Each of the authors of this brief editorial has their own personal Secher ‘anecdote’, and he remains a guiding light and constant source of inspiration. We have all borne witness to his impressive catheterization skills that have helped provide unique insight into local metabolite exchange kinetics across the brain, lungs, muscle, liver and kidneys, to help ‘better’ define key mechanisms (Bailey et al., 2017, 2018). But as a collective, we are unified through gentle ‘re‐education’ that during haemorrhage, heart rate (HR) is not invariably increased as traditionally described in many textbooks and emblazoned in the minds of emergency unit doctors, with potentially fatal consequences.

In a 1985 paper entitled ‘Vagal slowing of the heart during haemorrhage: observations from 20 consecutive hypotensive patients’ (Sander‐Jensen et al., 1986), a ‘tell‐tale’ table demonstrated that during the shock phase when the blood pressure was low, HR was also low (on average 73 beats/min) – not high as traditional dogma would have predicted. During the recovery phase when blood, albumin and crystalloids were supplied, and corresponding blood pressure increased, HR also increased (on average 102 beats/min). In a minireview (Secher & Bie, 1985), Niels H. Secher and Peter Bie emphasized how physiology textbooks incorrectly focus on the tachycardia associated with haemorrhagic shock, with such statements as ‘…the compensatory effects of the carotid sinus reflex, in response to the hypotension of intense peripheral vasoconstriction, and of tachycardia, are indisputable’.

In a follow‐up review (Secher et al., 1992), Niels H. Secher further outlined the complex changes in HR associated with hypovolaemic shock. He divided central hypovolaemia into three stages. The first stage describes a modest elevation in HR in response to a reduction (up to 15%) in central blood volume, but with further blood loss (up to 30%) HR decreases, and bradycardia prevails. Finally, as hypovolaemia continues, HR increases further and tachycardia develops. The important translational physiological ‘take‐home’ here is that these complex changes during hypovolaemia make it difficult to use HR for diagnostic purposes. Sadly, this complexity is still not fully appreciated or communicated in many of our modern‐day physiology textbooks.

This is but one example of Niels H. Secher's impact in the field of integrated translational human physiology. Much of his research has been published in *Experimental Physiology*, a journal that aligns perfectly with his clinical–scientific interests, and sporting prowess!

## AUTHOR CONTRIBUTIONS

Damian M. Bailey conceived the idea and with Christian Aalkjær wrote the first draft of the manuscript. Christian Aalkjær, Mads Fischer and Damian M. Bailey edited and revised the manuscript. All authors have read and approved the final version of this manuscript and agree to be accountable for all aspects of the work in ensuring that questions related to the accuracy or integrity of any part of the work are appropriately investigated and resolved. All persons designated as authors qualify for authorship, and all those who qualify for authorship are listed.

## CONFLICT OF INTEREST

D.M.B. is Editor‐in‐Chief of *Experimental Physiology*, Chair of the Life Sciences Working Group, member of the Human Spaceflight and Exploration Science Advisory Committee to the European Space Agency, member of the Space Exploration Advisory Committee to the UK Space Agency, member of the National Cardiovascular Network for Wales and South East Wales Vascular Network and is affiliated to the companies FloTBI, Inc. and Bexorg, Inc. focused on the technological development of novel biomarkers of brain injury in humans.

## FUNDING INFORMATION

D.M.B. is supported by a Royal Society Wolfson Research Fellowship (#WM170007).
